# Effectiveness of lanadelumab for preventing hereditary angioedema attacks: Subgroup analyses from the HELP study

**DOI:** 10.1111/cea.13974

**Published:** 2021-07-05

**Authors:** Douglas T. Johnston, Paula J. Busse, Marc A. Riedl, Marcus Maurer, John Anderson, Christina Nurse, Neil Inhaber, Ming Yu, Aleena Banerji, HELP Study Investigators, J. Hébert, B. Ritchie, G. Sussman, W. H. Yang, C. Escuriola Ettingshausen, M. Magerl, I. Martinez‐Saguer, M. Maurer, P. Staubach, S. Zimmer, M. Cicardi, F. Perego, M. A. Wu, A. Zanichelli, A. Al‐Ghazawi, M. Shennak, R. H. Zaragoza‐Urdaz, R. Ghurye, H. J. Longhurst, E. Zinser, J. Anderson, A. Banerji, A. P. Baptist, J. A. Bernstein, P. B. Boggs, P. J. Busse, S. Christiansen, T. Craig, M. Davis‐Lorton, S. Gierer, R. G. Gower, D. Harris, D. I. Hong, J. Jacobs, D. T. Johnston, E. S. Levitch, H. H. Li, R. F. Lockey, P. Lugar, W. R. Lumry, M. E. Manning, D. L. McNeil, I. Melamed, T. Mostofi, T. Nickel, W. R. Otto, A. A. Petrov, K. Poarch, C. Radojicic, S. M. Rehman, M. A. Riedl, L. B. Schwartz, R. Shapiro, E. Sher, A. M. Smith, T. D. Smith, D. Soteres, R. Tachdjian, H. J. Wedner, M. E. Weinstein, H. Zafra, B. L. Zuraw

**Affiliations:** ^1^ Carolina Asthma & Allergy Center Charlotte NC USA; ^2^ Division of Clinical Immunology and Allergy, Department of Medicine Icahn School of Medicine at Mount Sinai New York NY USA; ^3^ Division of Rheumatology, Allergy and Immunology University of California San Diego La Jolla CA USA; ^4^ Dermatological Allergology, Allergie‐Centrum‐Charité Department of Dermatology and Allergy Charité – Universitätsmedizin Berlin Berlin Germany; ^5^ Alabama Allergy & Asthma Center Birmingham AL USA; ^6^ Takeda Pharmaceutical Company Limited Lexington MA USA; ^7^ Division of Rheumatology, Allergy and Immunology, Department of Medicine Massachusetts General Hospital Harvard Medical School Boston MA USA

**KEY MESSAGES**

- Lanadelumab was effective compared with placebo in preventing HAE attacks across various patient subgroups.
- For all subgroups, lanadelumab 300 mg q2w reduced attack rates by >50% during the full treatment period.
- Attack reduction was >50% for most subgroups early on, with further reductions during steady state.

To the Editor:

Hereditary angioedema (HAE) is a rare disease that affects 1:50,000 people^1^ and is characterized by unpredictable attacks of angioedema affecting the skin and submucosal tissue.^1^, ^2^ Patients with HAE must have medication readily available to treat attacks, and may use long‐term prophylaxis (LTP) to prevent attacks.^1^ Approved therapeutics for LTP include the plasma kallikrein inhibitors lanadelumab and berotralstat; C1‐inhibitor (C1‐INH) replacement; and androgens.

Lanadelumab is approved in numerous countries for the prevention of HAE attacks in patients ≥12 years of age. The recommended starting dose is 300 mg every 2 weeks (q2w), but this may be reduced to 300 mg every 4 weeks (q4w) if patients are attack‐free for ≥6 months.^3^ Lanadelumab inhibits plasma kallikrein, thereby preventing the formation of bradykinin and the development of attacks.^4^ In the phase 3, randomized, double‐blind, placebo‐controlled hereditary angioedema long‐term prophylaxis (HELP) Study (NCT02586805), lanadelumab significantly reduced the occurrence of attacks over a 6‐month treatment period in patients ≥12 years of age with HAE type 1 or 2.^5^ Mean attack rates in patients treated with subcutaneous lanadelumab 300 mg q2w and 300 mg q4w decreased by 86.9% and 73.3%, respectively, versus placebo.

Given the wide heterogeneity in the clinical presentation of HAE,^2^ we evaluated the efficacy of lanadelumab in HELP in prespecified subgroup categories (age, sex, race, weight, body mass index (BMI), run‐in attack rate, HAE type, geographic region, prior LTP use and history of laryngeal attacks). A comparable reduction in attack rate among patients with respect to sex, BMI, run‐in attack rate and prior LTP use was reported previously.^5^ Here, we report findings for age, weight, HAE type and laryngeal attack history. Furthermore, results from a post hoc analysis that assessed efficacy before reaching steady state (days 0–69) and during steady state (days 70–182 as determined from the measured lanadelumab half‐life of 14 days^6^) are reported for all subgroups.

For the number of investigator‐confirmed attacks in each subgroup separately, each lanadelumab treatment group was compared with placebo using a Poisson regression model including the normalized run‐in period attack rate and treatment group as covariates, and accounting for potential overdispersion. *p*‐values for the comparisons were unadjusted and provided for exploratory purposes. Results are presented from a likelihood ratio test for the pairwise interaction of the subgroup with the treatment group using a Poisson regression model with fixed effects for treatment group, subgroup, subgroup by treatment group, and normalized baseline attack rate, and the logarithm of time (days) each patient was observed during the treatment period as an offset variable. Pearson's chi‐square scaling of standard errors was employed to account for potential overdispersion. All lanadelumab mean rate ratios relative to placebo are model based and compare the mean number of attacks per month (4 weeks). A ratio <1 indicates fewer attacks in the lanadelumab groups, and a ratio ≤0.5 indicates ≥50% reduction in attacks when compared with placebo.

A total of 125 patients received at least one dose of treatment (*n* = 28, 150 mg lanadelumab q4w; *n* = 29, 300 mg lanadelumab q4w; *n* = 27, 300 mg lanadelumab q2w; and *n* = 41, placebo) and were included in the analysis of the overall treatment period. The analysis of days 0–69 comprised 125 patients and the analysis of steady state comprised 120 patients (5 patients who discontinued before day 70 were excluded). Patients <18 years of age (*n* = 10) were excluded from the analysis of BMI subgroups.

Lanadelumab at each dose was effective in preventing HAE attacks during the overall treatment period (days 0–182) across the majority of subgroups for age, weight, HAE type and laryngeal attack history. For all subgroups with the 300 mg q2w dose, the mean rate ratios ranged from 0.069 to 0.377, and ratios for almost all subgroups with the 150 mg q2w and 300 mg q4w dose were <0.5 during the overall treatment period (Figure 1). The effectiveness of lanadelumab overall across these subgroups and subgroups for sex, BMI, run‐in attack rate and prior LTP use was also evident shortly after the initiation of treatment, with >50% attack rate reduction during days 0–69 for all evaluable subgroups except the subgroup with type 2 HAE (ratio 0.58, range 0.15–2.31) (Figure 2). Attack rates were further reduced during steady state, with >50% reduction for all evaluable subgroups.

**FIGURE 1:**
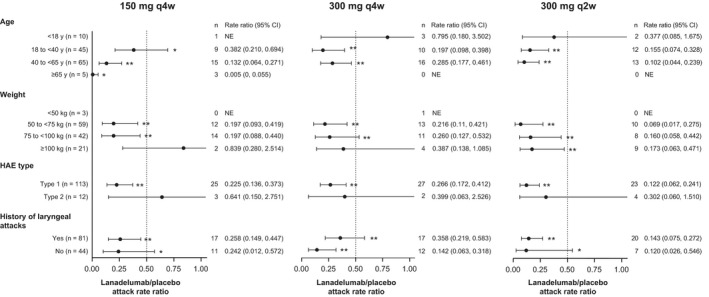
Reduction in baseline‐adjusted HAE attack rate vs. placebo during the overall treatment period (days 0–182) by subgroup. *N* for each overall subgroup category includes patients who received placebo. Mean (95% CI) rate ratios versus placebo are shown for each subgroup. *p < .05; **p < .001. HAE, hereditary angioedema; NE, not estimable; q2w, every 2 weeks; q4w, every 4 weeks

**FIGURE 2:**
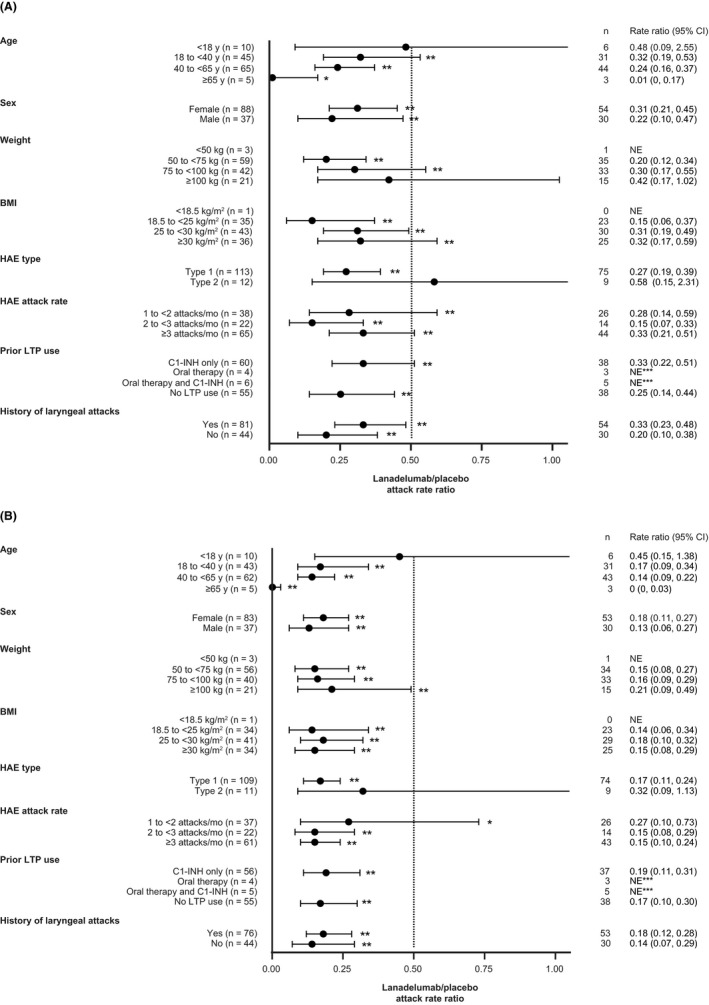
Reduction in HAE attack rate versus placebo for patients who received lanadelumab (150 mg q4w, 300 mg q4w or 300 mg q2w treatment groups combined) during days 0–69 (A) and during steady state (days 70–182) (B) by subgroup. N for each overall subgroup category includes patients who received placebo. Mean (95% CI) rate ratios vs. placebo are shown for each subgroup. *p < .05; **p < .001; ***the ratio was not estimable in the prior LTP oral and C1‐INH + oral subgroups because of the sparseness of the data. BMI, body mass index; C1‐INH, C1‐inhibitor; HAE, hereditary angioedema; LTP, long‐term prophylaxis; mo, month; NE, not estimable; q2w, every 2 weeks; q4w, every 4 weeks.

For the age subgroup interaction with the 150 mg q4w treatment group, *p* = .038, and for the history of laryngeal attacks interaction with the 300 mg q4w treatment group, *p* = .032. However, these *p*‐values should be interpreted with caution as they are not adjusted for multiple testing and are exploratory. The interaction of the weight subgroup with the 150 mg q4w and 300 mg q4w treatment groups was not estimable as the model was not able to converge with the interaction term in the model. The *p*‐values for all interactions during steady state were >.05.

Findings in several categories are notable. Firstly, patients' ages ranged from 12 to 73 years in this study, and although there were few patients in the <18 years and ≥65 years subgroups, there was a reduction in attacks when compared with placebo across age groups. Secondly, attack rate reductions were significant at approved doses regardless of disease activity, as defined by the frequency of attacks during the run‐in period.^5^ Thirdly, lanadelumab was effective regardless of past LTP use; mean rate ratios relative to placebo were <0.3 for the overall treatment period,^5^ <0.5 during days 0–69, and <0.3 during steady state, not only for patients who previously used C1‐INH for LTP but also for patients who did not use LTP. Finally, a consistent response across body weight and BMI subgroups at approved doses was observed, suggesting that dose adjustment of lanadelumab by weight is not required. This is in agreement with a pharmacokinetic/pharmacodynamic and exposure‐response analysis, which showed that despite higher exposure to lanadelumab in patients with lower body weight, intrinsic covariates including age, sex and baseline disease activity had no effect on the pharmacokinetic parameters of lanadelumab, and the pharmacodynamic response to treatment was comparable across body weight groups.^7^ Furthermore, the safety and tolerability of lanadelumab as indicated by adverse event profiles^5^, ^6^ were not affected by weight‐dependent exposure differences and were consistent across all other subgroups (data not shown).

The variability and unpredictability of HAE, and the impact of external factors on the onset of attacks, means that individualized care is important and necessitates the evaluation of treatments across patient and disease characteristics. Although the data were less robust for subgroups of extremes of age and weight, owing to small sample sizes, this analysis did not identify any specific subgroups with increased or decreased benefit from approved doses of lanadelumab treatment, suggesting consistent efficacy of lanadelumab in all patients receiving HAE prophylactic therapy. Results from other clinical studies, including the HELP open‐label extension study^8^ and an ongoing study in patients aged 2 to <12 years,^9^ will further elucidate the impact of patient and disease characteristics on the treatment effect of lanadelumab and any need for dosing adjustments in specific populations.

## CONFLICTS OF INTEREST

DTJ has received consulting/speaker fees from CSL Behring, Pharming and Shire (a Takeda company); and consulting fees from BioCryst and Regenxbio. PJB reports grants from Shire (a Takeda company); a lecture honorarium from CVS Health; and consulting fees from BioCryst, CSL Behring, Pearl Therapeutics, Pharming and Shire (a Takeda company). MAR has received research grants from BioCryst, CSL Behring, Pharming and Shire (a Takeda company); consulting fees from Adverum, Attune, BioCryst, CSL Behring, KalVista, Pharming, Pharvaris and Shire (a Takeda company); speaker honoraria from CSL Behring, Pharming and Shire; and is a medical advisory board member of the US Hereditary Angioedema Association. MM has received research grant support and/or speaker/consultancy fees from Adverum, Attune, BioCryst, CSL Behring, KalVista, Pharming, Pharvaris and Shire (a Takeda company). JA is a speaker bureau member for CSL Behring, Pharming, Biocryst and Shire (a Takeda company); has received consultancy fees from CSL Behring, Pharming and Shire (a Takeda company); and is a clinical trial investigator for Pharming, KalVista, BioCryst, CSL Behring and Shire (a Takeda company). CN, NI and MY are full‐time employees of Takeda Pharmaceutical Company Limited and hold stock/stock options in Takeda. AB has received institutional research/study support from BioCryst and Shire (a Takeda company) and/or honoraria for consulting from BioCryst, CSL Behring, KalVista, Pharming, Pharvaris and Shire (a Takeda company).

## AUTHOR CONTRIBUTIONS

DJT and MM made substantial contributions to the conception and design of the study, the acquisition of data, and the analysis and interpretation of data. PJB, MAR, JA and AB were involved in the acquisition and interpretation of data. CN, NI and MY were involved in the analysis and interpretation of data. All authors read and provided critical input on the manuscript, and approved the final manuscript. Although employees of the Sponsor were involved in the design, collection, analysis, interpretation and fact‐checking of information, the content of this manuscript, the interpretation of the data, and the decision to submit the manuscript for publication in *Clinical & Experimental Allergy* was made by the authors independently.

## ETHICAL APPROVAL

The HELP study was conducted in accordance with Good Clinical Practice guidelines and the principles of the Declaration of Helsinki as well as other applicable local ethical and legal requirements. All patients or caregivers provided written informed consent (or assent from patients <18 years) at screening.

## DATA AVAILABILITY STATEMENT

The redacted study protocol and redacted statistical analysis plan for the HELP study have been previously published.^5^ The data sets, including individual participants’ data supporting the results of the study, will be made available after the publication of study results within 3 months from initial request, to researchers who provide a methodologically sound proposal. The data will be provided after its de‐identification, in compliance with applicable privacy laws, data protection and requirements for consent and anonymization.

## APPENDIX 1

HELP Study investigators: Canada: J. Hébert, B. Ritchie, G. Sussman, W. H. Yang; Germany: C. Escuriola Ettingshausen, M. Magerl, I. Martinez‐Saguer, M. Maurer, P. Staubach, S. Zimmer; Italy: M. Cicardi, F. Perego, M. A. Wu, A. Zanichelli; Jordan: A. Al‐Ghazawi, M. Shennak; Puerto Rico: R. H. Zaragoza‐Urdaz; United Kingdom: R. Ghurye, H. J. Longhurst, E. Zinser; United States: J. Anderson, A. Banerji, A. P. Baptist, J. A. Bernstein, P. B. Boggs, P. J. Busse, S. Christiansen, T. Craig, M. Davis‐Lorton, S. Gierer, R. G. Gower, D. Harris, D. I. Hong, J. Jacobs, D. T. Johnston, E. S. Levitch, H. H. Li, R. F. Lockey, P. Lugar, W. R. Lumry, M. E. Manning, D. L. McNeil, I. Melamed, T. Mostofi, T. Nickel, W. R. Otto, A. A. Petrov, K. Poarch, C. Radojicic, S. M. Rehman, M. A. Riedl, L. B. Schwartz, R. Shapiro, E. Sher, A. M. Smith, T. D. Smith, D. Soteres, R. Tachdjian, H. J. Wedner, M. E. Weinstein, H. Zafra, B. L. Zuraw.
